# Prenatal imaging of upper urinary tract abnormalities: when is MRI useful?

**DOI:** 10.1007/s00247-025-06465-2

**Published:** 2025-11-28

**Authors:** Stasianne M Mallin, Monica M Forbes-Amrhein, Megan B Marine

**Affiliations:** Department of Radiology and Imaging Sciences, Indiana University, Riley Children’s Health, IU Health Medical Group, 705 Riley Hospital Drive, Room 1053, Indianapolis, IN 46202 USA

**Keywords:** Urinary tract dilatation, Urogenital abnormalities, Cystic renal dysplasia, Fetal kidneys, Magnetic resonance imaging, Ultrasound

## Abstract

Upper urinary tract abnormalities are commonly encountered on fetal imaging. While ultrasound (US) remains the primary imaging modality for evaluation of the fetal kidneys, magnetic resonance imaging (MRI) can play a significant complementary role in the diagnosis and management of these patients with upper urinary tract abnormalities. This paper will review the most common prenatal pathologies encountered in the kidneys, including urinary tract dilatation (UTD), renal agenesis, renal ectopia, multicystic dysplastic kidney (MCDK), and other cystic renal diseases, and will include a discussion of when and why fetal MRI should be considered in these diagnoses. Images from prenatal US and fetal MRI examinations will be included.

**Graphical Abstract:**

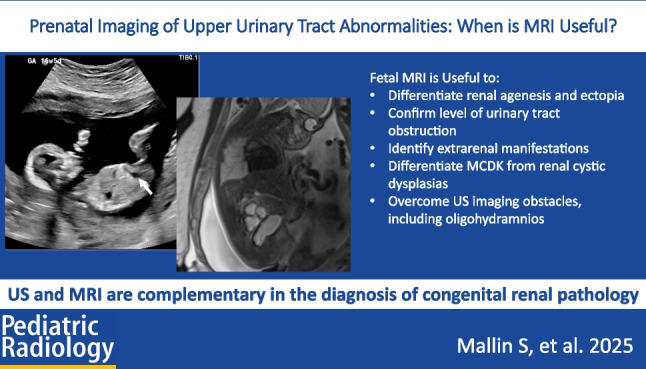

## Introduction

Congenital abnormalities of the kidneys and urinary tract account for approximately 25% of all prenatal anomalies [1]. The abnormalities range greatly in severity, with some having no consequence to the fetus, while the most severe result in high mortality rates. These defects may be due to genetic, epigenetic, and/or environmental factors. Specific prenatal exposures can result in urinary tract abnormalities including maternal medications (angiotensin-converting enzyme inhibitors, angiotensin-receptor blockers), nutritional deficiencies (examples include folate, iron), and other maternal conditions such as diabetes, obesity, and illicit drug exposure [1]. Ultrasound (US) is typically the first screening imaging modality used to assess fetal anatomy allowing for evaluation of the fetal urinary tract. Based on the results of the prenatal US exam, fetal magnetic resonance imaging (MRI) may be indicated for additional evaluation [2].

### Ultrasound evaluation of the fetal urinary tract

**Fig. 1:**
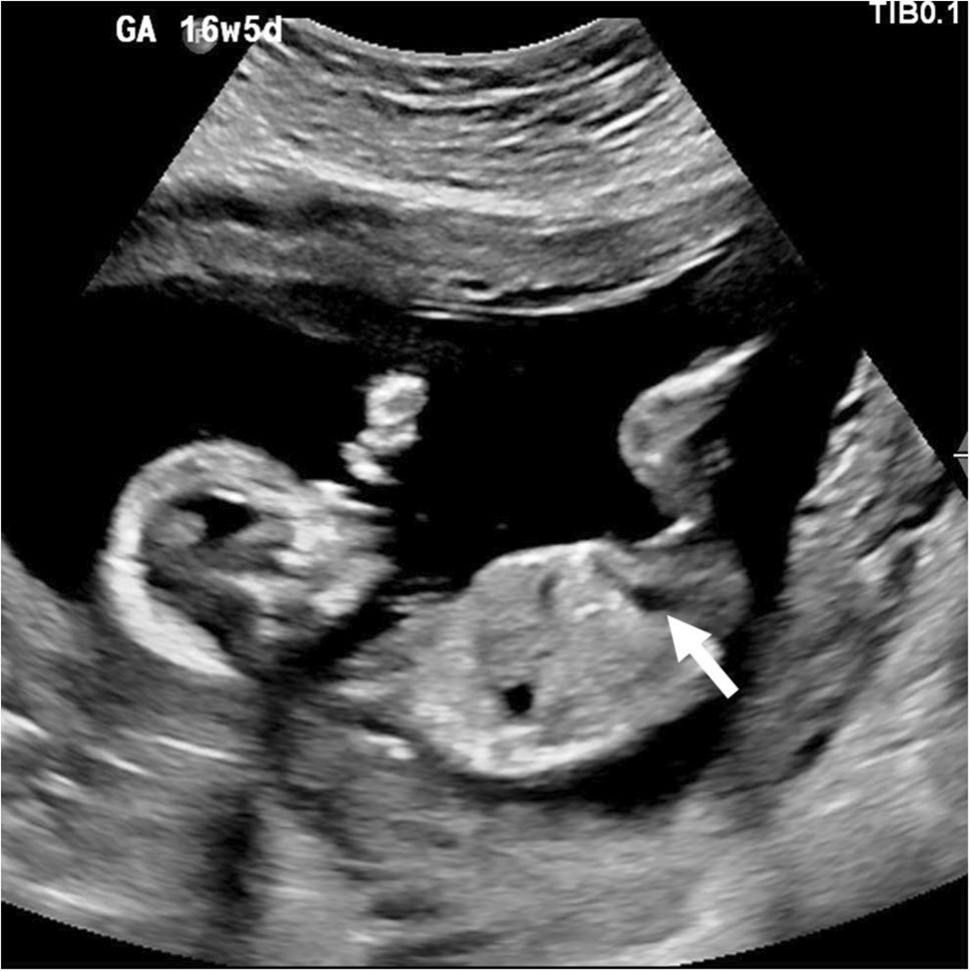
Sagittal ultrasound of a 16-week gestation male fetus showing the anechoic fetal urinary bladder (*arrow*)

**Fig. 2:**
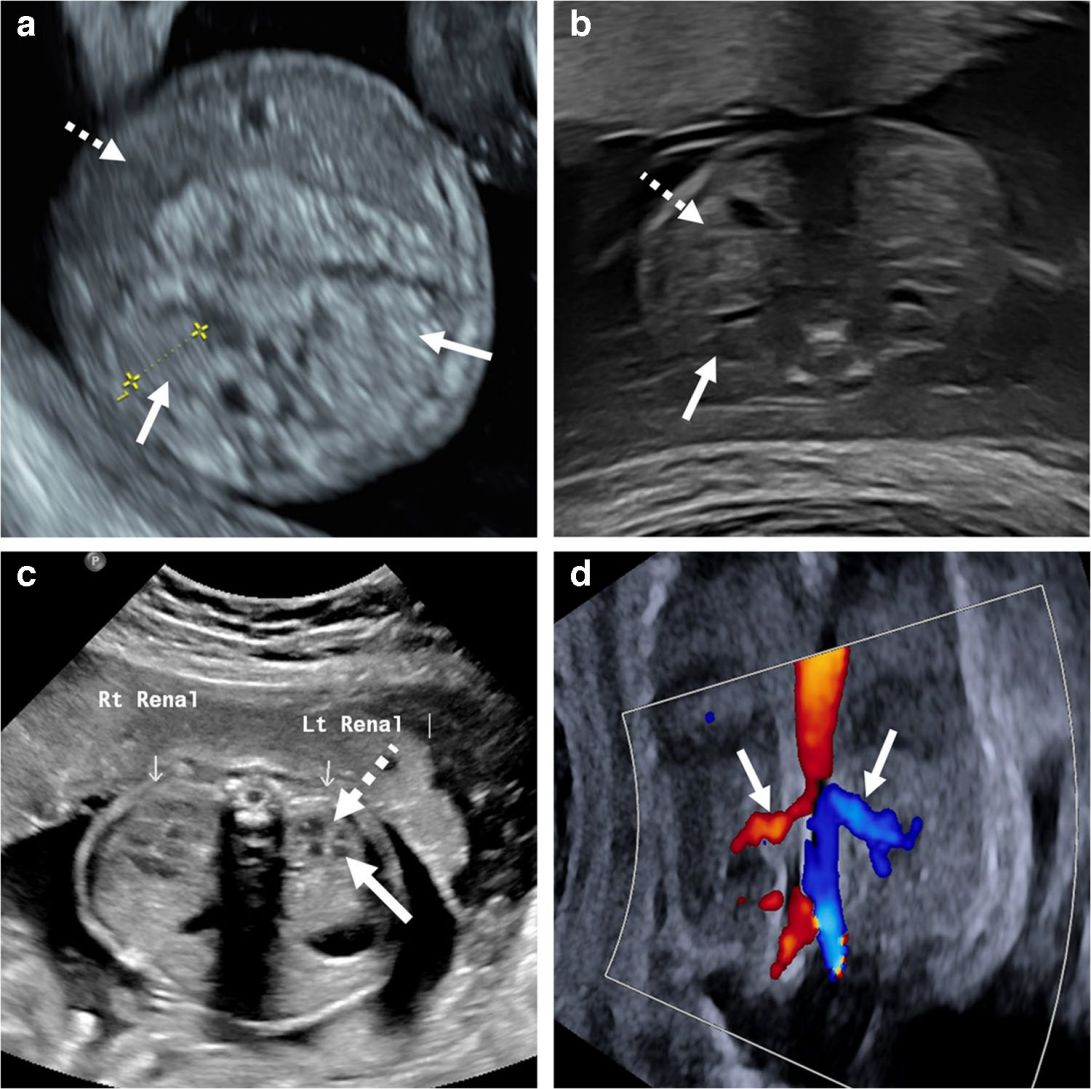
Fetal ultrasound of the kidneys with progressing gestation. **a** Axial ultrasound of a 15-week gestation male fetus showing that the renal parenchyma (*arrow*) is echogenic compared to the liver (*dotted arrow*). **b** Axial ultrasound of a 20-week gestation male fetus showing that the renal parenchyma (*arrow*) is now hypoechoic compared to the liver (*dotted arrow*). **c** Axial ultrasound of a 28-week gestation male fetus showing that the renal medulla (*arrow*) decreases in echogenicity becoming hypoechoic compared to the cortex (*dotted arrow*). **d** Coronal Doppler ultrasound of a 28-week gestation male fetus showing the renal arteries (*arrows*)

First trimester US may be obtained, often for dating purposes, but the routine anatomy US scan should occur at approximately 20 weeks’ gestation. US is used to identify the presence, location, size, structure, and echogenicity of the kidneys, in addition to the presence and size of the urinary bladder, and amniotic fluid volume. The fetal kidneys begin producing urine as early as 10 weeks’ gestation [1]. At 11–12 weeks’ gestation, the fetal bladder can be visualized (Fig. 1) [2, 3]. Amniotic fluid is formed by secretions of the placenta, fetal membrane, and skin until 10 weeks’ gestation, after which fetal urine production is the major source of amniotic fluid. Therefore, oligohydramnios secondary to renal pathology is typically not present until the time of the routine 20-week anatomy US [2]. The renal sizes are directly proportional to gestational age (Renal length in cm=number of gestation weeks×1.1 cm) [2, 3]. The range of normal lengths for gestation is variable, and reference charts are available with data from 14 through 42 weeks’ gestation [4]. Kidneys appear echogenic in early gestation, gradually becoming hypoechoic in comparison to the adjacent bowel and liver (Fig. 2a, b). The renal cortex is hyperechoic compared to the medulla (Fig. 2c). The renal pelvises appear anechoic in the medial aspect of transverse sections [5]. The renal arteries can be identified with Doppler imaging [2]. Normal ureters should never be visualized antenatally [2]. At 34 weeks’ gestation, the nephron completes development. Neonatal kidneys measure approximately 4–4.5.5 cm in longest dimension at birth in full-term infants [1, 4].

### MRI technique in evaluation of the fetal urinary tract

While US is the primary imaging modality in the evaluation of the fetal urinary tract, fetal MRI has been shown to complement the prenatal US regarding specification of fetal urinary tract abnormalities, often modifying the US diagnosis and impacting therapeutic approaches [5, 6]. MRI can overcome obstacles that make US challenging, such as oligohydramnios, fetal lie, and maternal obesity [3]. Additionally, the larger fields of view offered by MRI allow for visualization of the entire urinary tract, particularly in the coronal and sagittal planes, allowing for precise evaluation of the position of the kidneys and ureteral courses.

MRI is typically performed in the second or third trimester and relies heavily on T2-weighted imaging for evaluation of the urinary tract. Typical fetal MRI protocols include 3–5-mm slice spacing using T2 Single-Shot Fast Spin Echo (SSFSE) and T2 Steady-State Free Precession (SSFP), maternal breath hold T1 gradient echo imaging (GRE), diffusion-weighted imaging (DWI), and echoplanar imaging (EPI) [7]. SSFSE and SSFP are both relatively resistant to fetal motion and provide detailed images of the entire fetal anatomy. At some institutions, 3-D sequences are added to the protocol, creating images with an MR urography appearance aiding in the evaluation of the ureters and level of obstruction or other pathology. Techniques reported include T2 50-mm T slab coronal imaging as well as 3-D True Fast Imaging with Steady-State Free Precession (TRUFI) [5].

DWI is helpful in identifying fetal kidneys, particularly when renal agenesis or ectopic kidney is being considered, as the renal parenchyma results in high DWI signal [8]. T1-weighted images are particularly useful in differentiating dilated ureters from surrounding bowel loops. Meconium-filled bowel is T1 hyperintense, whereas the urine-filled ureters are T1 hypointense [8]. In the setting of urinary tract abnormalities associated with oligohydramnios, MRI can also be used to estimate total lung volumes as assessment for pulmonary hypoplasia. The observed to expected total fetal lung volume ratios can be calculated using reference lung volumes by Meyers et al. [9]. While current literature indicates that lung volumes alone do not predict outcomes in fetal genitourinary abnormalities, this value may be helpful in fetuses in which there is a discrepancy between lung volume and amniotic fluid levels, such as a fetus with normal amniotic fluid but less than expected total fetal lung volume ratio [10]. Future studies are needed to better assess lung volumes in predicting outcomes in fetal genitourinary abnormalities.

## Common pathology in the fetal upper urinary tract

### Urinary tract dilatation

Urinary tract dilatation (UTD) in the fetus is very common, found in 1–2% of prenatal US studies [11]. Most causes of prenatally identified UTDs (70–80%) are transient or physiologic. The remaining etiologies include a spectrum of pathologies such as ureteropelvic junction (UPJ) obstruction, ureterovesical junction (UVJ) obstruction, vesicoureteral reflux (VUR), complicated duplex kidneys, and lower urinary tract obstruction [3, 12]. The 2014 Multidisciplinary UTD classification system is one of the most widely used systems to classify the severity of prenatal UTD, correlating it with the risk of postnatal uropathies. The classification utilizes the anterior-posterior renal pelvic diameter (APRPD) measured on US, in addition to calyceal dilation, renal parenchymal thickness and appearance, ureteral dilation, and bladder anomalies [12, 13]. The optimum US measurement of the APRPD is obtained when the spine is closest to the transducer, and the measurement is made of the maximum intrarenal diameter of the renal pelvis in the transverse plane of the kidney [12, 13]. APRPD measurements and grading of UTD, as well as follow-up imaging recommendations, are found in Table 1 [6, 8, 12, 14]. Postnatally, the UTD classification system (graded P1-P3) is also utilized and has been shown to predict the need for surgical intervention and urinary tract infection risk [6, 14].

Fetal MRI is useful for UTD evaluation, particularly if there is associated oligohydramnios. MRI can provide detailed information on the ureteral course and caliber to detect the level of urinary tract obstruction. MRI is also useful for assessing the presence of bladder pathology and associated malformations [3, 13]. The classification and grading of UTD is the same for US and MRI [15]. Common causes of fetal UTD are reviewed below.

**Table 1:** Updated classification and imaging follow-up recommendations for prenatal urinary tract dilatation (UTD) based on anterior-posterior renal pelvic diameter (APRPD) on ultrasound [12, 13]. Antenatal classification is divided into three categories: normal, UTD A1, and UTD A2-3. UTD A1 is considered low-risk for postnatal complications; UTD A2-3 is considered an increased risk for developing urinary tract infections, requiring VUR imaging or surgical intervention [12]. Note that any dilation of calyces is classified as UTD A2-3. The presence of ureter dilation, abnormal renal parenchyma, abnormal bladder, or oligohydramnios when APRPD is ≥4 mm is also automatically classified as UTD A2-3 [12]

	Normal	UTD A1	UTD A2-3
APRPD 16–27 weeks	<4 mm	4–7 mm	≥7 mm
APRPD ≥28 weeks	<7 mm	7–10 mm	≥10 mm
Follow-up	None	1 additional fetal US at ≥32 weeks	Follow-up fetal US in 4–6 weeks

#### Ureteropelvic junction obstruction

**Fig. 3:**
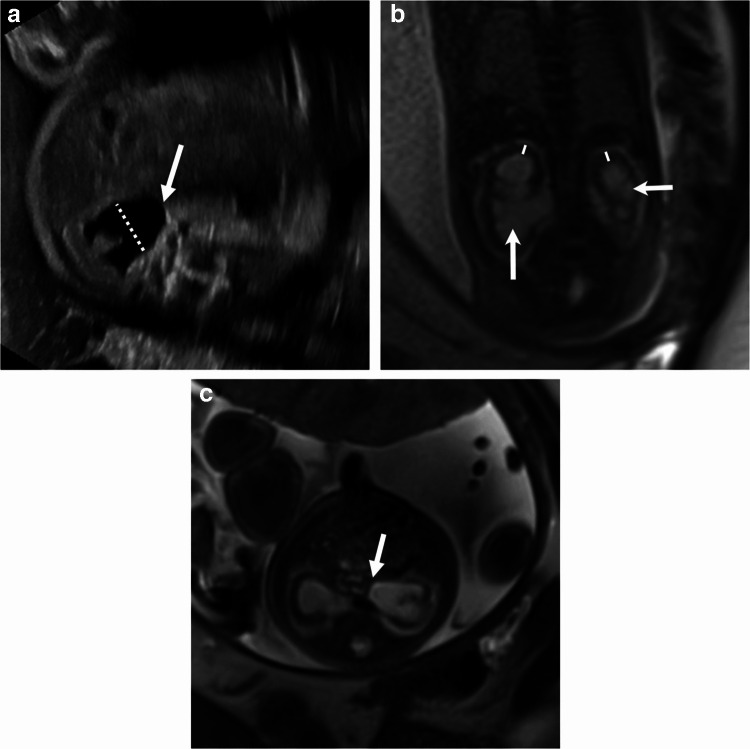
Patient with bilateral ureteropelvic junction obstruction. **a** Axial ultrasound of a 30-week gestation male fetus showing an enlarged anterior-posterior diameter (*dotted line*) of the right kidney, dilatation of the pelvis and calyces (*arrow*), and non-visualization of the ureter. **b** Coronal Single-Shot Fast Spin Echo (SSFSE) MRI images obtained the same day revealing dilation of the bilateral renal collecting system (*arrows*) with diffuse thinning of the renal cortex (*line*). The ureters were not visualized. **c** Axial SSFSE image showing the dilatation of the left renal pelvis that tapers abruptly at the junction with the ureter (*arrow*)

Ureteropelvic junction obstruction is the most common cause of prenatal UTD, accounting for approximately 41% of cases [14]. Prenatal US usually demonstrates unilateral, rarely bilateral, pelvicalyceal dilation without ureteral dilation or bladder abnormality (Fig. 3a) [2, 11]. The prognosis of UPJ obstruction is favorable, as there is poor correlation between the degree of pelvic dilatation and postnatal renal function [3]. However, if cystic obstructive dysplasia develops, impaired postnatal renal function is more likely. In the case of cystic dysplasia, US may demonstrate a thinned echogenic cortex containing cortical cysts. Third trimester US can help assess for the progression of the obstruction [14].

Fetal MRI is a useful adjunct to US in the setting of UPJ obstruction. If there is concern for bilateral UTD, MRI can assess for the presence of a lower urinary tract obstruction (Fig. 3b, c). In severe UPJ obstruction, rupture of the renal calyces and urine extravasation can occur, leading to an intrarenal cystic lesion or perirenal fluid collection on US. MRI is useful in such circumstances to evaluate for and confirm perirenal urinoma or urinary ascites [3]. If obstructive cystic dysplasia develops, MRI is sensitive to those changes which appear as increased T2-hyperintense signal of the parenchyma and/or discrete cortical cysts [3].

#### Ureterovesical junction obstruction

Ureterovesical junction obstruction typically presents with unilateral hydronephrosis, megaureter (ureteral dilation), and normal appearance of the bladder [11]. On US, UVJ obstruction should be considered in the setting of megaureter; however, megaureter is not specific as vesicoureteral reflux can also result in secondary megaureter [3, 16]. Urinary tract dilatation may increase in utero but usually decreases progressively after birth [3]. Prenatal follow-up scans are required to follow amniotic fluid volume stability, especially if there is bilateral renal pathology or ureterocele [3].

**Fig. 4:**
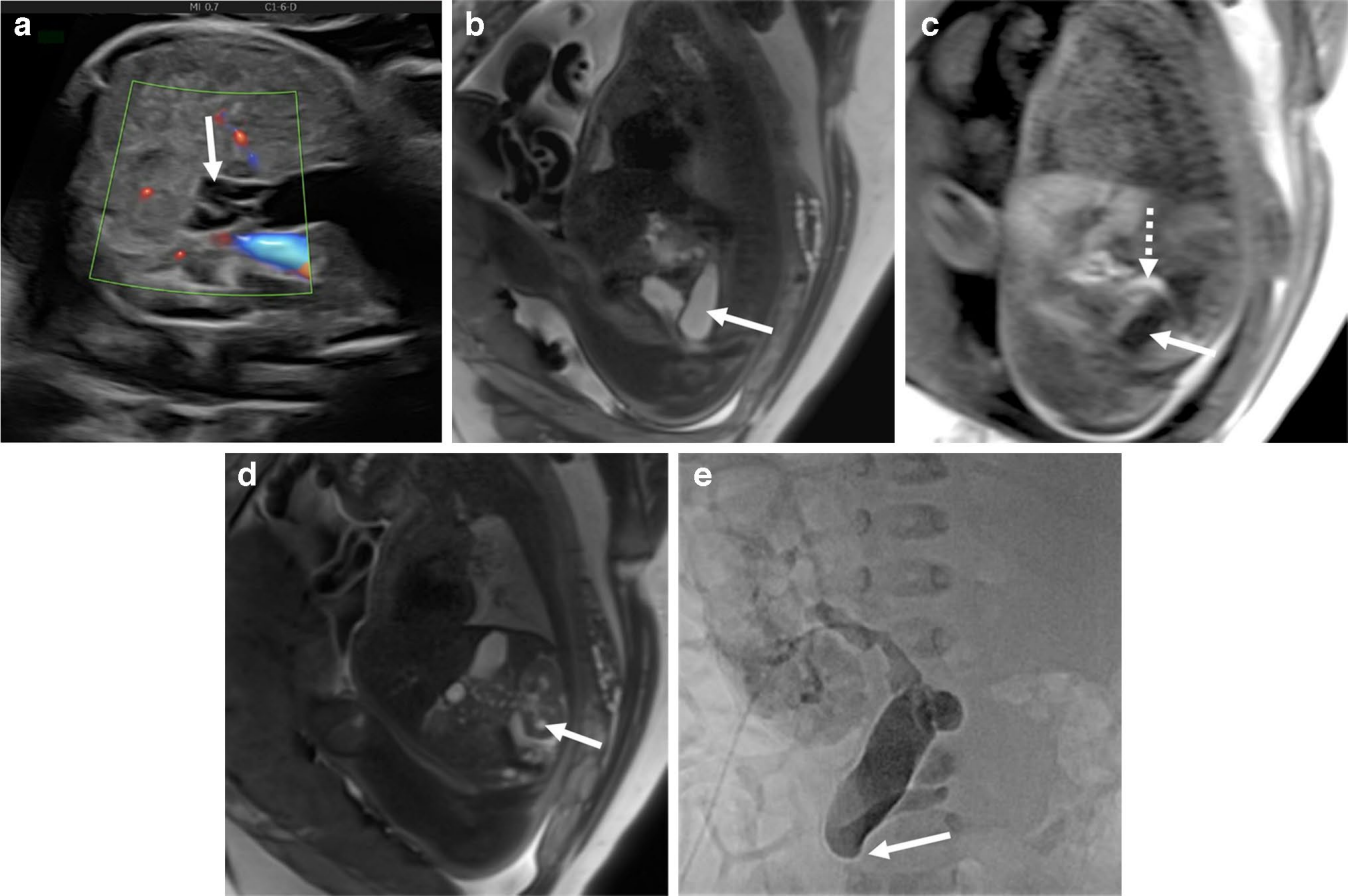
Patient with ureterovesical junction (UVJ) obstruction. **a** Axial ultrasound of a 29-week gestation male fetus demonstrating a tortuous, tubular anechoic abdominal structure (*arrow*) without internal Doppler flow, consistent with a dilated ureter.** b** Sagittal Single-Shot Fast Spin Echo (SSFSE) MRI obtained the same day demonstrating a T2-hyperintense tortuous dilated distal ureter (*arrow*). **c** Sagittal T1 MRI showing T1-hypointense signal within the tubular structure (*arrow*) confirming this to be the ureter, not bowel. Note the T1-hyperintense signal within the adjacent bowel loops (*dotted* *arrow*). **d** Sagittal SSFSE MRI showing that fetal renal collecting system dilation is only mild (*arrow*). **e** Postnatal percutaneous nephrogram in a coronal oblique plane confirms the diagnosis of a UVJ obstruction with beaking of the distal ureter (*arrow*) and absent flow of contrast into the urinary bladder

On US, bowel may be distinguished from the dilated ureter by attempting to follow the ureteral course from the kidney to the bladder (Fig. 4a), but this can often be challenging [3]. MRI can be useful to evaluate megaureter (Fig. 4b) [3]. Additionally, MRI can often demonstrate the distal ureteral insertion site and assess for underlying associations, such as duplex kidney and/or ureterocele [3]. MRI easily distinguishes T1-hypointense megaureter from adjacent T1-hyperintense bowel loops, secondary to meconium signal (Fig. 4c) [3]. Complications such as urinoma and cystic obstructive dysplasia are less common in the setting of UVJ obstruction than UPJ obstruction but may occur (Fig. 4d) [3]. Postnatal imaging can confirm the presence of UVJ obstruction (Fig. 4e). A high proportion spontaneously resolves in early childhood, particularly if the diameter of the ureter is less than 8 mm [17].

#### Vesicoureteral reflux

Vesicoureteral reflux accounts for 7–35% of prenatal UTD [14]. VUR can be unilateral or bilateral and is associated with duplex collecting systems, which may demonstrate ectopic insertion of the distal ureter (see “Renal duplication” below). VUR can present with or without a dilated ureter [11]. Additionally, the degree of UTD affecting the renal pelvis and ureter can vary at different moments in a single US examination, offering clues to the diagnosis [3, 11]. US may demonstrate impaired renal growth as well as thickening of the walls of the collecting system, but the appearance of VUR is otherwise not specific, making it difficult to diagnose with confidence prenatally with either US or fetal MRI [16]. The diagnosis is only reliably distinguishable from obstructive pathology with postnatal imaging, most commonly voiding cystourethrogram or voiding urosonography [16, 18]. The vast majority of VUR resolves by 2 years of age [3].

### Renal duplication

**Fig. 5:**
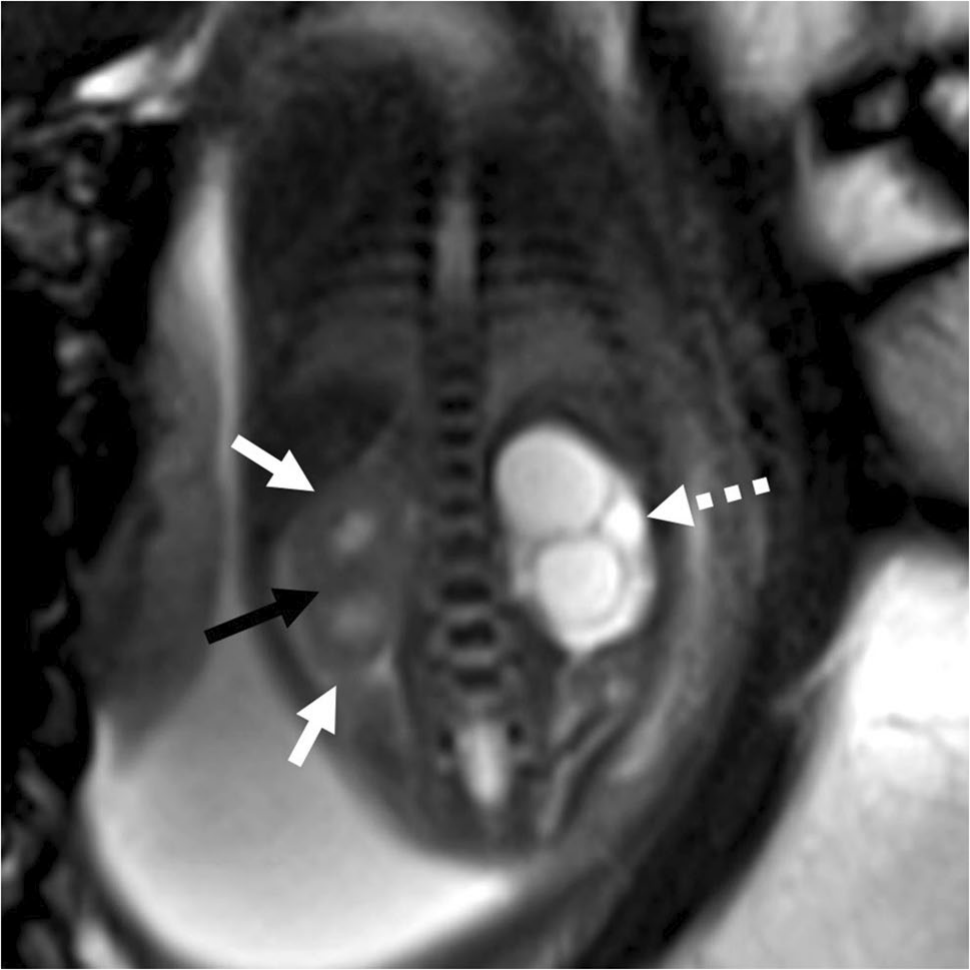
Coronal Single-Shot Fast Spin Echo (SSFSE) images of a 25-week female fetus showing a right renal duplication with separate upper and lower pole collecting systems (*white arrows*) with intervening parenchymal bar (*black arrow*). Also note a concurrent left multicystic dysplastic kidney (*dotted arrow*)

Renal duplication can affect one or both kidneys and encompasses a range of developmental differences. An incomplete duplex kidney is defined as duplication of the renal pelvis with two ureters that join before draining into the bladder. A complete duplex kidney consists of a kidney with two separate collecting systems; each moiety has its own distinct ureter that inserts into the bladder independently (Fig. 5) [3].

A duplex kidney may be identified on US, particularly if there is dilation of one or both moieties and/or if the renal size is large or asymmetrically larger than expected for gestational age. Imaging will demonstrate two collecting systems separated by a band of cortex at the mid kidney. Ureteral dilation may or may not be present. A duplex kidney may be considered a normal variant; however, it can be associated with urinary tract pathology, as defined by the Weigert-Meyer rule, which depicts ureteral obstruction and dilation of the upper pole moiety and VUR (with or without dilation) of the lower pole moiety [19]. Ureteral obstruction of the upper pole moiety may be due to an ectopic distal ureteral insertion, which is identified on US by following the dilated ureter from the kidney into the fetal pelvis [20]. Ectopic ureteral insertion can occur anywhere along the genitourinary tract, but the inferior medial bladder remains the most common site [21]. The ectopic ureter may have an associated ureterocele (dilation of the distal ureter that is contained within the bladder wall) which can result in mechanical obstruction of the distal ureter [21]. A large ureterocele can also obstruct the contralateral ureter or even prolapse into the urethra (cecoureterocele), causing complete bladder outlet obstruction and subsequent oligohydramnios [3]. It is important to examine the bladder several times during US imaging to avoid mistaking a ureterocele in a decompressed bladder for a normal bladder. The fetal bladder should empty and fill every 30 min in the second and third trimester (more frequently in the first trimester), so US evaluations may require 40 min or more [22].

**Fig. 6:**
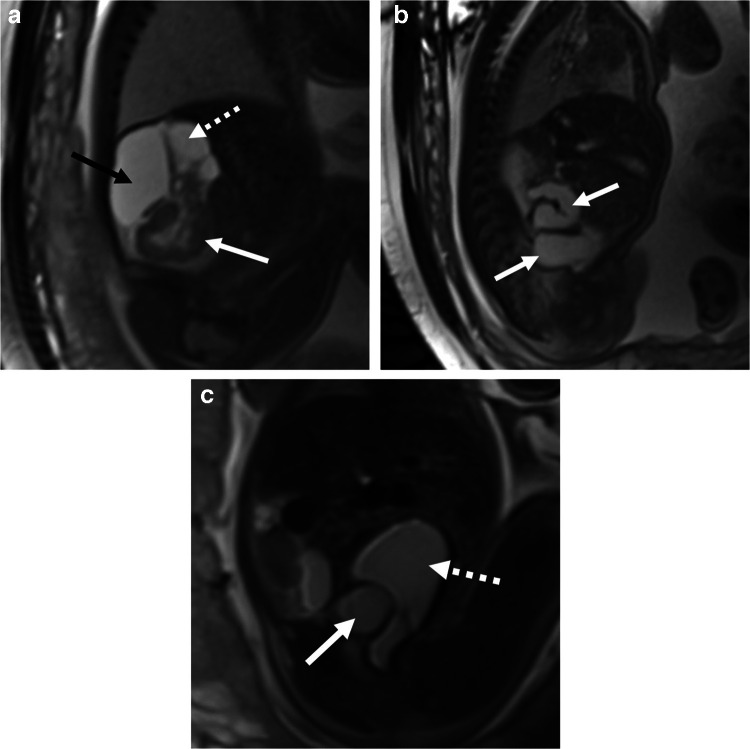
MRI imaging of a 30-week female fetus demonstrating a duplex kidney with upper pole moiety obstruction. **a** Sagittal Single-Shot Fast Spin Echo (SSFSE) image showing normal renal parenchyma at the lower pole moiety (*white arrow*), dilation of the upper pole collecting system with cystic obstructive dysplasia of the parenchyma (*dotted white arrow*), and a larger posterior fluid collection, likely urinoma (*black arrow*). **b** Sagittal SSFSE image showing the dilated, tortuous upper pole ureter (*white arrows*). **c** Coronal SSFSE image showing a ureterocele (*white arrow*) at the ureter insertion into the urinary bladder (*dotted arrow*)

MRI should be considered in cases in which the anatomic evaluation on US is unclear. MRI can demonstrate the duplex parenchyma, the course of the dilated ureter, evaluate for the site of ectopic insertion, and assess for the presence of a ureterocele (Fig. 6a-c). Additionally, MRI should be considered when there is concern for contralateral renal abnormality or complication, such as a urinoma in the setting of contralateral ureteral obstruction. As with other causes of urinary obstruction, renal parenchymal cystic dysplasia can develop, which may be better characterized on MRI. Of note, MRI can often demonstrate the appearance of duplex renal parenchyma in the absence of pelvic dilation, typically considered an incidental finding in the absence of any associated UTD [3].

### Renal agenesis

**Fig. 7:**
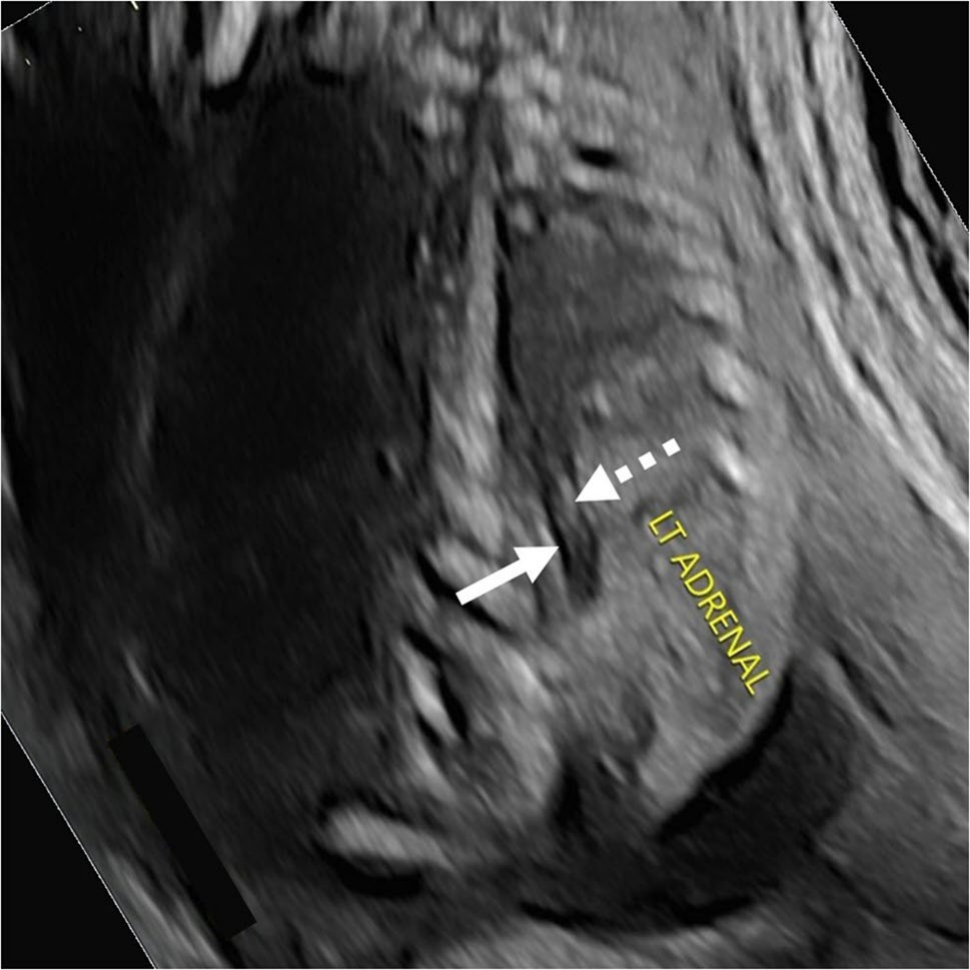
Coronal ultrasound imaging of a 23-week female fetus with left renal agenesis demonstrating the abnormal configuration of the adrenal gland, which has a laying, linear configuration, rather than the usual inverted “Y” configuration. Notice the hypoechoic cortex (*white arrow*) and hyperechoic (*dotted white arrow*) medulla. The adrenal gland should be distinguished from the diaphragm (not shown), which is uniformly hypoechoic

**Fig. 8:**
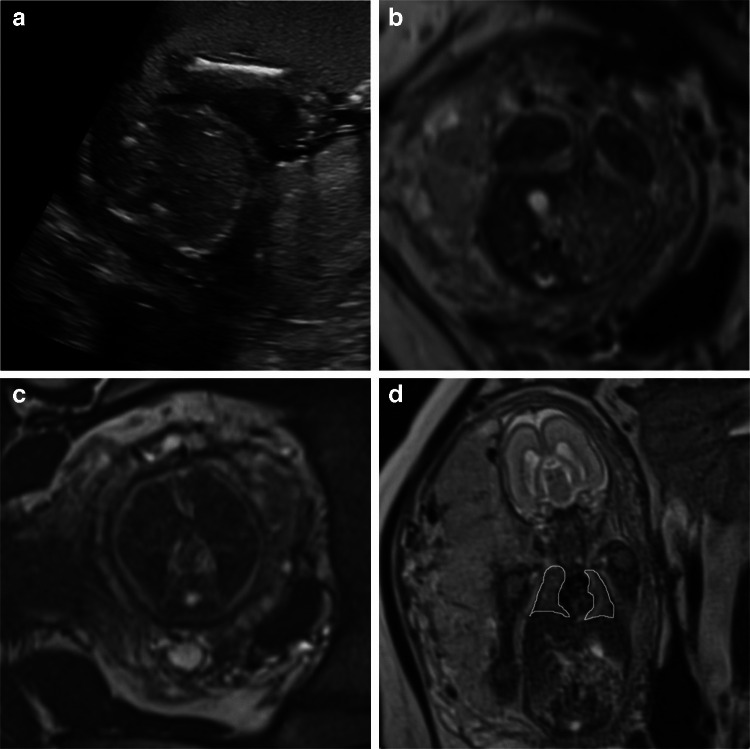
**a** Axial ultrasound of a 20-week gestation male fetus showing anhydramnios and absent bilateral kidneys. **b** MRI imaging obtained the same day confirming absent kidneys, bilaterally, on the axial Single-Shot Fast Spin Echo (SSFSE) image. Note the absence of amniotic fluid. **c** Axial SSFSE image at the level of the pelvis confirming non-visualized urinary bladder. **d** Coronal SSFSE image demonstrating Potter’s Ssequence with compression of the fetus and T2-hypointense hypoplastic lungs (white outline)

Renal agenesis is the congenital absence of one or both kidneys. Unilateral renal agenesis itself does not impact fetal development; however, up to 30% are associated with genetic syndromes or other anomalies, including contralateral renal pathology including vesicoureteral reflux, UPJ obstruction, or UVJ obstruction [23, 24]. Evaluation of the adrenal gland morphology can be useful if considering the diagnosis of renal agenesis. In renal agenesis (or ectopic kidney), the ipsilateral adrenal gland will appear elongated, linear, and “lying down” (Fig. 7) [3]. Care must be taken to not mistake the crus of the diaphragm for an adrenal limb. The diaphragm will be of uniform echogenicity compared to the adrenal gland which will contain an echogenic medulla and hypoechoic cortex. A normal adrenal gland, however, does not exclude renal absence as renal dysplasia and subsequent involution can occur after the initial normal adrenal gland development [25]. Bilateral renal agenesis is uniformly fatal without intervention. Oligohydramnios and/or anhydramnios develop after the 10th week of gestation when the placenta stops producing amniotic fluid and urine is not being produced due to the absence of functional renal tissue. US can be very challenging with non-visualization of the kidneys, absent renal arteries on Doppler, and absence of the urinary bladder (Fig. 8a) [9].

MRI is particularly helpful in suspected bilateral renal agenesis, given the oligohydramnios/anhydramnios which significantly limits US evaluation. MRI can confirm the absence of renal parenchyma (Fig. 8b), with DWI being a valuable sequence. The presence of an ectopic kidney should be excluded. The persistently empty bladder can be confirmed on T2-weighted sequences. Rarely, a patent urachus may retro fill the bladder and should be considered if the bladder is visible but the kidneys cannot be identified [3]. MRI is also helpful in demonstrating the associated Potter sequence traits in the setting of oligohydramnios, which includes a small appearing chest, pulmonary hypoplasia, club feet, low-set ears, and abnormal facies (Fig. 8c, d, and later discussed in Fig. 13) [26]. The Potter sequence is not specific to bilateral renal agenesis and can be seen in any pathology resulting in severe oligohydramnios or anhydramnios. Total fetal lung volume measurements can also be obtained on MRI, but as discussed above, its prognostic value in genitourinary pathology may be most helpful in fetuses in which there is a discrepancy between lung volume and amniotic fluid levels [10].

### Renal ectopia

Renal ectopia is defined as the abnormal location of one or both kidneys. The severity ranges from a morphologic normal kidney abnormally located to complex ectopia with fusion anomaly (such as horseshoe kidney or crossed-fused renal ectopia). Again, the ipsilateral adrenal gland of the affected side may appear elongated or linear, but this is not seen in all cases of renal ectopia [25]. The urinary bladder and amniotic fluid volume should be normal assuming no additional renal tract pathology.

**Fig. 9:**
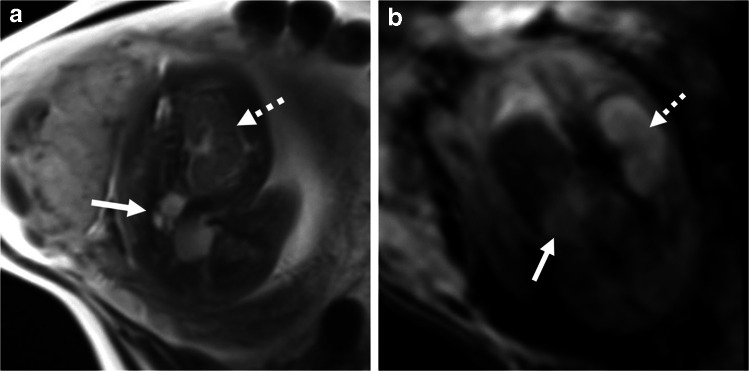
**a** Fetal MRI of a 29-week gestation male fetus. Oblique coronal Single-Shot Fast Spin Echo (SSFSE) images demonstrate an ectopic right multicystic dysplastic kidney (MCDK) (arrow) located in the pelvis and the orthotopic position of the left kidney (dotted arrow). **b** Oblique coronal diffusion-weighted imaging (DWI) showing restricted diffusion of the pelvic tissue (arrow) confirming this as renal tissue. The left kidney is also showing restricted diffusion (dotted arrow)

Ectopic kidneys are most commonly located in the pelvis and tend to be small and/or malrotated (Fig. 9a, b) [3]. Both crossed ectopia (without fusion) and cross-fused ectopia can occur. Renal ectopia can be difficult to diagnose prenatally on US and may present as an empty renal fossa with concern for unilateral renal agenesis [27]. In cross-fused ectopia, the axes of both fused kidneys can be either parallel to each other or at right angles in the hemiabdomen. Cross-fused ectopic kidneys are usually more inferior in location than orthotopic kidneys [3]. The most common fusion pattern is with the lower pole of the normally located kidney fused to the superior pole of the ectopic/cross-fused kidney. Left to right ectopy is three times more common than right to left [27]. The length of the visualized renal parenchymal bulk is often greater than would be expected for a single kidney, potentially approaching twice the normal length for gestational age. US Doppler may identify both renal arteries in the hemiabdomen [20]; however, unless there is a high pre-test probability, this may be overlooked as a normal variant of multiple renal arteries.

Horseshoe kidney occurs in 1 in 400 individuals, making it the most common congenital abnormality of the upper urinary tract [20]. US may show an oblique position of both renal lower poles which converge towards the midline. Visualization of renal parenchymal corticomedullary differentiation within the connecting isthmus confirms the presence of a horseshoe kidney [3]. In a horseshoe kidney, ureters should be assessed for an anomalous course or dilatation [8].

**Fig. 10:**
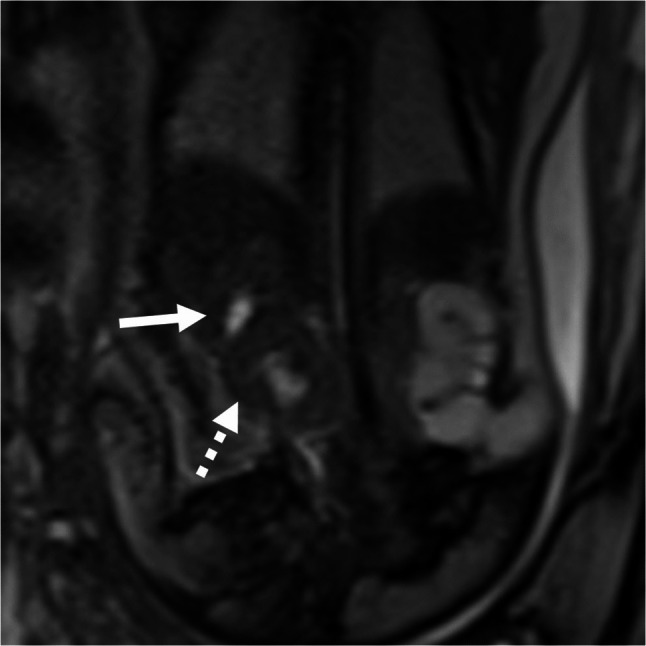
Coronal Single-Shot Fast Spin Echo (SSFSE) MRI of a 29-week gestation male fetus showing a left-to-right crossed fused renal ectopia (right - arrow, left - dotted arrow). The right and left renal moieties are positioned at 90° and both are located in the right hemiabdomen

Fetal MRI should be considered in the case of apparent unilateral renal agenesis as ectopic renal parenchyma may be present. T2 and DWI sequences can be very useful in identifying ectopic renal parenchyma (Fig. 9b). Fetal MRI can better delineate cross-fused ectopia (Fig. 10), horseshoe kidney, and ureteral course. It is important to correctly identify ectopic renal parenchyma given the increased occurrence of vesicoureteral reflux related to the atypical positioning of the ureter(s) and subsequent dysplastic changes. Additionally, ectopic kidneys are also at increased risk for trauma in childhood and beyond, making it important to confirm this diagnosis as it could impact future considerations [24].

### Simple renal cyst

Simple renal cysts are typically unilateral, solitary, and nongenetically transmitted, ranging in size from a few millimeters to several centimeters. On US, simple renal cysts will be anechoic and well circumscribed. Posterior acoustic enhancement, while a common feature of postnatal simple cysts, is typically not appreciated on prenatal imaging [3]. There are no septa or calcifications, differentiating the lesion from a cystic renal tumor [3]. True simple renal cysts are rare and most resolve by 24 weeks gestation [3]. Follow-up prenatal US in 4–6 weeks is recommended to assess for new cysts but continued follow-up is not required after confirmation of a single simple cyst [3]. The majority of prenatally diagnosed simple renal cysts are asymptomatic and rarely require treatment [3].

Fetal MRI should be considered if the cyst is located in the upper pole of the kidney, as this may actually represent an obstructed and dilated upper pole moiety of a duplex kidney. MRI can evaluate for the appearance of the duplex kidney, dilated ureter, and other associations such as ureterocele. Additionally, if there is a family history of cystic renal disease, MRI may be considered as hereditary cystic renal diseases may begin as an apparent simple renal cyst on US but may be more complex on MRI. Additionally, MRI will allow for a more sensitive evaluation of the contralateral kidney.

### Cystic renal diseases

Cystic renal diseases can be subdivided into multicystic dysplastic kidney (MCDK) versus other cystic renal dysplasia including TCF2 gene anomalies, single gene and multigene ciliopathies, and obstructive cystic dysplasia.

#### Multicystic dysplastic kidney

Multicystic dysplastic kidney is the most common incidental cystic renal lesion on prenatal US [28]. MCDK is defined as multiple, noncommunicating cysts of various sizes replacing the renal parenchyma with the absence of normal intervening renal parenchyma [29]. In MCDK, the normal tubular branching of the renal collecting system fails to develop. MCDK is usually detected on second trimester US with the appearance of multiple anechoic, noncommunicating cysts without normal renal parenchyma or a visible renal pelvis. A unilateral MCDK may be accompanied by a contralateral hypertrophic kidney due to compensatory overgrowth. Pathologies associated with MCDK include VUR, UPJ obstruction, and renal fusion anomalies [30]. MCDK will decrease in size, involuting, over time. If this occurs in utero or soon after birth, it can be mistaken for renal agenesis [3]. In unilateral MCDK, the bladder volume and amniotic fluid should be normal, assuming normal function of the contralateral kidney [30]. US can accurately diagnose MCDK and can be used to guide antenatal counseling. MCDK is not typically associated with genetic disorders; however, MCDK can be seen in the setting of some syndromes or sequences (i.e., VACTERL syndrome) [30]. Segmental MCDK may occur in either moiety of a duplex kidney. If an upper pole has a multicystic appearance, there should be a high suspicion of a duplicated collecting system with chronically obstructed upper pole moiety and evaluation for a dilated ureter and associated ureterocele can help to make the correct diagnosis [20].

**Fig. 11:**
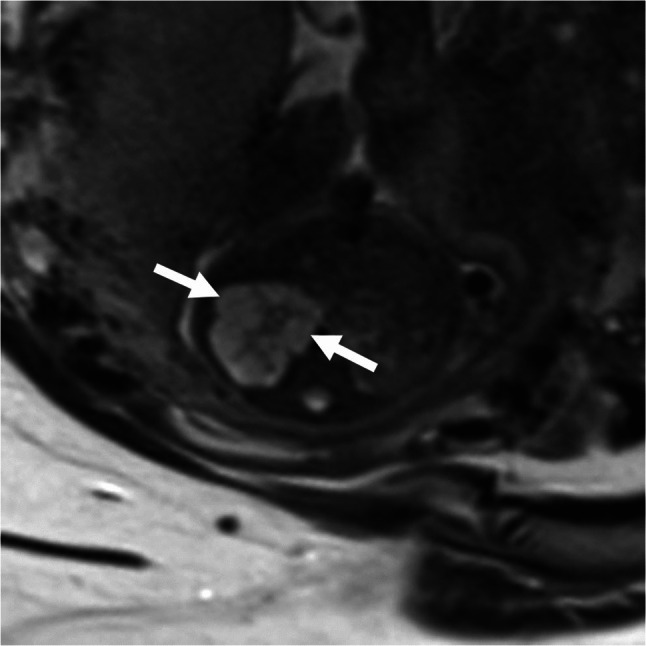
Axial Single-Shot Fast Spin Echo (SSFSE) MRI of a 25-week gestation female fetus with a right multicystic dysplastic kidney. Note the cysts of differing sizes (arrows) replacing the renal parenchyma

Fetal MRI can add additional diagnostic information in the assessment of MCDK. MRI will reveal T2-hyperintense cysts replacing the bilateral renal parenchyma with minimal parenchyma identified (Figs. 5 and 11) [3, 30]. On fetal MRI, MCDK should not have a renal pelvis or branching collecting system [30], often confirmed on the coronal imaging, and differentiating MCDK from UTD or other cystic renal diseases. Low renal parenchymal signal on DWI may suggest a lack of functioning renal tissue, helping to confirm the diagnosis and differentiate it from UTD [8]. One series demonstrated the vast majority (47/51) of cases of unilateral MCDK had low signal on DWI on MRI, aiding in diagnosis [31]. In the same series, MRI proved useful by correctly changing the diagnosis in 12 cases, initially thought by US to be either renal agenesis, ectopic kidney, or UTD. In this series, MRI also demonstrated extrarenal central nervous system anomalies in 3/51 cases and other urinary tract anomalies in 16/51 cases, most commonly ipsilateral ectopic kidney [31]. MRI is obviously very useful in the setting of bilateral MCDK given severe oligohydramnios and can help to confirm this diagnosis [3].

Prenatal US surveillance should be conducted after the diagnosis of unilateral MCDK to monitor the contralateral kidney’s appearance and amniotic fluid volume [29]. Given the contralateral kidney is functioning as a solitary kidney, it must be closely monitored for the development of UTD, particularly given the association with contralateral obstructive pathology. Postnatal renal US should be performed shortly after birth to confirm the diagnosis of unilateral MCDK and to importantly evaluate the contralateral single functioning kidney [29].

#### Other cystic renal dysplasia

Cystic renal dysplasia is important to differentiate from MCDK given the difference in etiology and natural history [30]. Kidneys affected by cystic renal dysplasia may have a variety of appearances on US ranging from increased echogenicity of the renal parenchyma, loss of corticomedullary differentiation, and/or presence of cysts [30]. Reversal of corticomedullary differentiation on prenatal US has also been reported in cases of cystic renal dysplasia, amongst other nephropathies [32]. The presence of any identifiable renal parenchyma differentiates cystic renal dysplasia from MCDK, as does the identification of a central renal collecting system (renal pelvis and calyces) [30]. Detailed anatomic evaluation of the entire fetus is critical given the variety of underlying etiologies and associated pathology. Identification of additional anomalies can help assist with prenatal diagnosis and understanding postnatal prognosis. The postnatal function of the kidneys is dictated by the underlying etiology, with different degrees of renal function and related problems (such as hypertension) on postnatal evaluation [30]. Genetic etiologies of cystic renal dysplasia include TCF2 (also called HNF1B) gene anomalies and ciliopathies (both single and multigene ciliopathies). Previously described secondary obstructive cystic renal dysplasia can also occur from longstanding UPJ or lower urinary tract obstruction.

**Fig. 12:**
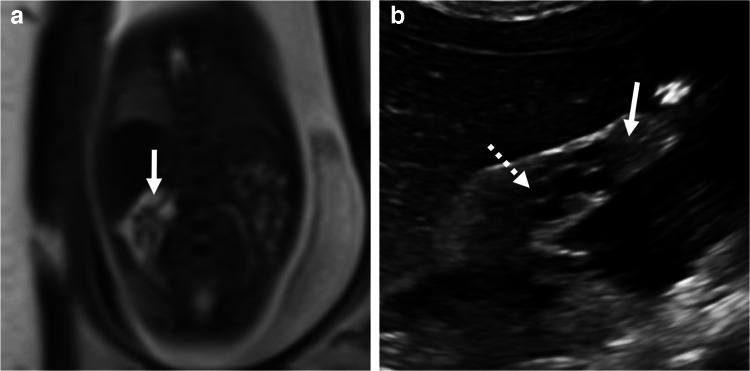
**a** Coronal Single-Shot Fast Spin Echo (SSFSE) MRI of a 24-week gestation male fetus with postnatal confirmation of a TCF2/HNF1B gene mutation (renal cysts and diabetes syndrome) showing right cystic renal dysplasia and absent left kidney. The right kidney is small with increased T2 signal of the renal parenchyma and numerous small parenchymal cysts (arrow). **b** Longitudinal renal ultrasound at day one of life showing a small echogenic right kidney with poor corticomedullary differentiation (arrow) and multiple medullary cysts (dotted arrows)

Anomalies of the TCF2 gene which provides instructions for making the hepatocyte nuclear factor-1 beta (HNF1B) protein cause renal cysts and diabetes syndrome (RCAD). RCAD has been reported to be a main cause of fetal renal hyperechogenicity, as well as the second most common etiology of cystic kidney diseases in childhood [33]. The clinical manifestations include renal cysts, pancreatic anomalies (resulting in diabetes mellitus), and abnormalities of the liver and genital tract. RCAD is also referred to as maturity-onset diabetes of the young (MODY) type 5 [33]. While antenatal cysts may also be present, the phenotype is variable, and the main prenatal presentation is bilateral hyperechoic kidneys [34]. The majority of RCAD will have normal amniotic fluid volume and normal-sized kidneys [35]. A recent case series of three fetuses demonstrated reversal of kidney cortico-medullary differentiation on fetal MRI suggesting this may point to the specific genetic condition, distinguishing it from other causes of hyperechoic kidneys on fetal ultrasound (Fig. 12a) [33]. Fetal MRI can also demonstrate extrarenal manifestations with pancreatic agenesis and hypoplasia reported in the fetus [36]. The postnatal imaging appearance of the kidneys can vary, with subcapsular, corticomedullary, and/or medullary cysts being a major feature (Fig. 12b).

**Fig. 13:**
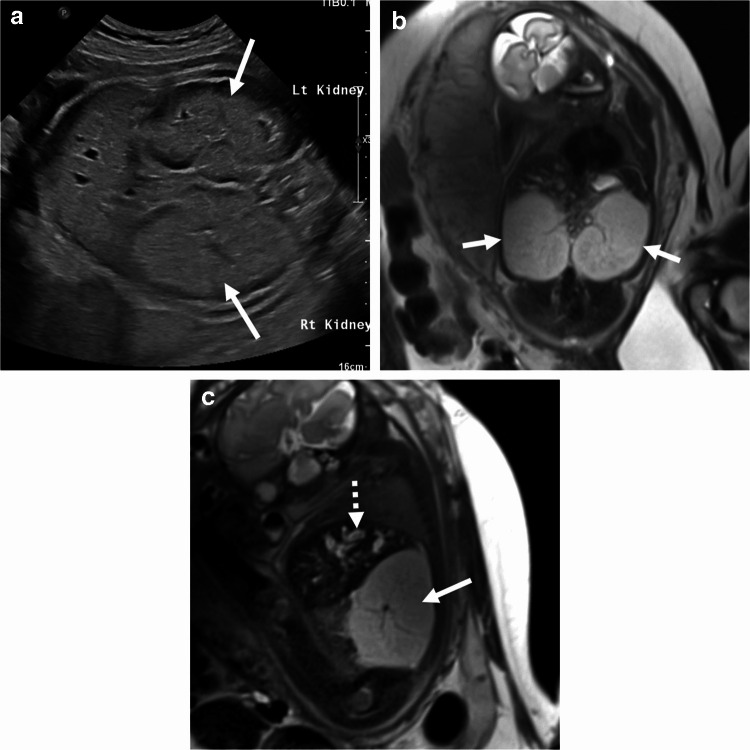
**a** Coronal fetal US of a 30-week gestation age male fetus with autosomal recessive polycystic kidney disease (ARPKD) showing bilateral enlarged kidneys with diffuse increased echogenicity (arrows). **b** Coronal Single-Shot Fast Spin Echo (SSFSE) MRI images obtained the same day showing massively enlarged bilateral kidneys (arrows) with diffuse increased T2 signal without discrete cysts. Small appearing chest and hypoplastic lungs are also noted as part of Potter’s sequence. **c** Sagittal SSFSE MRI showing enlarged, T2- hyperintense kidney (arrow). Caroli’s disease is also present as evidenced by cystic dilation of multiple intrahepatic bile ducts with central dot sign (dotted arrow)

Ciliopathies result from a defect in the primary cilia/centrosome complex and have many implications for various organs. The kidney defect is associated with cysts and renal failure. These are subdivided into single and multigene ciliopathies [30]. Single gene ciliopathies include autosomal recessive polycystic kidney disease (ARPKD), autosomal dominant polycystic kidney disease (ADPKD), Von Hippel-Lindau, and oral-facio-digital syndrome. In the authors’ experiences, ARPKD is the most common prenatally diagnosed single gene ciliopathy. ARPKD presents with exceptionally large kidneys with innumerable tiny cysts. The microcysts may not be discernable on US and the renal parenchyma will appear diffusely echogenic (Fig. 13a) or with reversed corticomedullary differentiation [32]. Oftentimes, severe oligohydramnios accompanies ARPKD, making US a challenging modality for assessment of fetal abnormalities [5].

Fetal MRI is particularly useful in confirming ARPKD given the accompanying oligohydramnios. MRI can reveal the large kidneys and the presence of microcysts in the renal parenchyma. The large kidneys increase the abdominal circumference and can superiorly displace the diaphragms, extrinsically compressing the chest and contributing to pulmonary hypoplasia, which can again be evaluated using MRI (Fig. 13b) [30]. ARPKD is also associated with Caroli’s disease type II, also known as Caroli’s syndrome which results in small bile duct dilatation of the liver and congenital hepatic fibrosis [37]. Fetal MRI can demonstrate the “central dot” sign which has been well described postnatally, demonstrating intrahepatic biliary duct dilation, and in association with the abnormal kidneys, can confirm the diagnosis (Fig. 13c) [38]. The bile duct dilation has also recently been described on prenatal US as diffuse tubular structures arranged in a “horn comb” sign in the liver [37]. Fifty percent of patients with ARPKD in the perinatal period will succumb to pulmonary hypoplasia and renal failure, while those that survive will require a renal transplant in childhood [3].

**Fig. 14:**
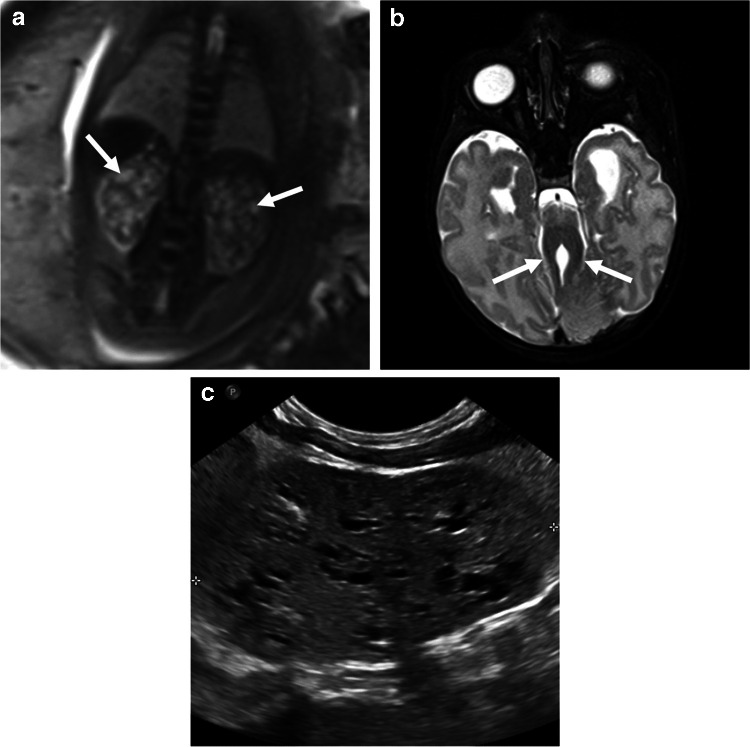
**a** Coronal Single-Shot Fast Spin Echo (SSFSE) MRI of a 31-week gestation female fetus with postnatally confirmed Joubert syndrome, a ciliopathy, demonstrating cystic renal dysplasia with bilateral innumerable T2- hyperintense renal cysts (arrows). **b** Axial T2 with fatsaturation MRI of the postnatal brain shows elongated superior cerebellar peduncles (arrows) resulting in the molar tooth appearance typical of Joubert syndrome. **c** Postnatal longitudinal ultrasound showing the dysplastic appearance of the left kidney. The right kidney (not shown) had a similar appearance

Multigene ciliopathies resulting in cystic renal dysplasia have widely variable phenotypes and are quite complex [30]. Fetal MRI is extremely important to evaluate for associated abnormalities in cases of suspected renal cystic dysplasia as identifying additional abnormalities can significantly impact and narrow the differential diagnosis and help guide prenatal genetic testing and counseling. See Table 2 for a list of more commonly encountered multigene ciliopathy syndromes and associated extrarenal abnormalities, many of which can be identified on fetal MRI. Figure 14.

**Table 2:** Various multigene ciliopathy syndromes with cystic kidneys and renal failure with the included associated extrarenal abnormalities that may be seen

	Neurologic	Gastrointestinal	Ophthalmic	Musculoskeletal	Other
Senior Loken syndrome			Retinitis pigmentosa		
Joubert syndrome (Fig. 14a-c)	Cerebellar vermian hypoplasia “Molar tooth sign” Neurocognitive delay Ataxia		Retinal coloboma	Polydactyly	Dysmorphic facies
COACH syndrome (subtype of Joubert syndrome)	Same as Joubert syndrome (see above)	Hepatic fibrosis	Retinal coloboma		Hypertelorism Ptosis
Meckel-Gruber syndrome	Occipital encephalocele Microcephaly	Hepatic fibrosis		Polydactyly	Congenital heart defects Lethal
Bardet-Biedl syndrome	Neurocognitive delay		Retinitis pigmentosa	Polydactyly	Obesity Hypogonadism in males
Zellweger spectrum disorders [39]	Neurocognitive delay, seizures, sensorineural deafness	Hepatomegaly and hepatic dysfunction, cholestasis, biliary dysgenesis	Pigmentary retinopathy, cataracts	Chondrodysplasia punctata	Dysmorphic facies

## Conclusion

If identified with prenatal imaging, congenital abnormalities of the upper urinary tract are often associated with good clinical outcomes, particularly if the renal disease is isolated to one kidney. While US is the primary imaging technique, fetal MRI adds valuable information in the evaluation of many commonly encountered upper urinary tract abnormalities and should be considered when pathology of the kidney is identified. Fetal MRI can often allow for improved prenatal diagnosis, ensuring appropriate prenatal counseling and planning for postnatal management.

## Data Availability

No datasets were generated or analysed during the current study.
